# Frost Tolerance Under Cold Flooding Involves the Presence of Specific PR Proteins and Is Linked to Snow Mould Pathogen Resistance

**DOI:** 10.3390/ijms27177514

**Published:** 2026-08-22

**Authors:** Ewa Pociecha, Barbara Jurczyk, Jana Moravčíková, Zuzana Gerši, Ewa Dubas, Anna Janeczko

**Affiliations:** 1 Department of Plant Breeding, Physiology and Seed Science, Faculty of Agriculture and Economics, University of Agriculture in Krakow, Podłużna 3, 30-239 Cracow, Poland; barbara.jurczyk@urk.edu.pl; 2 Faculty of Natural Sciences, Institute of Biology and Biotechnology, University of Ss. Cyril and Methodius in Trnava, Námestie Jozefa Herdu 2, 917 01 Trnava, Slovakia; jana.moravcikova@ucm.sk (J.M.); zuzana.gersi@ucm.sk (Z.G.); 3 The Franciszek Górski Institute of Plant Physiology, Polish Academy of Sciences, Niezapominajek 21, 30-239 Cracow, Poland; e.dubas@ifr-pan.edu.pl (E.D.);

**Keywords:** frost tolerance, flooding stress, β-1,3-glucanase, glutathione, sucrose synthase (SuS), sucrose phosphate synthase (SPS), snow mould, winter rye

## Abstract

Winter survival of crops is influenced by multiple environmental stress factors, including low temperature, excessive soil moisture, and pathogen pressure. With climate change, the role of flooding during cold acclimation has become increasingly important. This study examined the effect of cold flooding on frost tolerance of two winter rye (*Secale cereale* L.) lines differing in resistance to snow mould. Plants were subjected to cold acclimation for three weeks at 4 °C, followed by ten days of flooding at the same cold temperature. Cold flooding improved frost tolerance only in the snow mould-resistant line (343), which maintained the presence of a 50 kDa β-1,3-glucanase isoform and exhibited a flooding-induced decrease in cytosolic sucrose synthase (SuS) activity in leaves, while maintaining higher SuS activity in roots. In contrast, the less resistant line (620) lacked the 50 kDa isoform in roots and showed substantially lower SuS activity than line 343; however, leaf SuS activity increased during prolonged flooding to levels comparable with those of line 343. The 35 kDa isoform was no longer detectable in the leaves of either line after one or ten days of flooding, whereas in the roots of both lines, its accumulation was maintained after one day but was abolished after ten days of flooding. Additionally, line 620 exhibited prolonged flooding-induced upregulation of *glu*-8 and *TLP*3 genes, which, in the absence of protein accumulation, suggests post-transcriptional or translational constraints associated with prolonged stress. These findings demonstrate that higher frost tolerance under cold flooding involves the accumulation of specific pathogenesis-related (PR) proteins, maintaining a high reduced and oxidised glutathione ratio and metabolic adjustment of sucrose synthesis. Furthermore, cold acclimation alone did not differentiate frost tolerance between rye genotypes differing in snow mould resistance, whereas flooding under low-temperature conditions enhanced freezing tolerance only in the resistant line, indicating that flooding may function as a positive signal involved in frost tolerance induction.

## 1. Introduction

Winter hardiness is a critical trait for crop survival in temperate climates. Previous research has focused mainly on low-temperature stress and cold acclimation processes that enable plants to develop frost tolerance through physiological and molecular adjustments. However, climate change has introduced new challenges, including increased episodes of soil flooding in winter due to snowmelt and rainfall. Flooded soils create hypoxic or anoxic conditions, alter metabolic pathways, and increase susceptibility to snow mould (*Microdochium nivale*), a fungal pathogen of winter cereals. Moreover, responses to low- and high-temperature flooding, including changes in the photosynthetic apparatus and carbohydrate metabolism, are distinct, which suggests the involvement of different regulatory mechanisms [1]. Plants exposed to cold-induced flooding undergo oxygen deprivation in the root zone, triggering anaerobic metabolism and the accumulation of anaerobic metabolites such as ethanol [2]. These metabolic shifts can impair cellular functions and reduce plant tolerance to stress conditions. One of the typical adaptive responses shared by both abiotic stresses, such as low temperature, and biotic stresses, such as pathogen attack, is the induction of pathogenesis-related (PR) proteins. Structural modelling has revealed ice-binding surfaces (IBS) in some PR enzymes, namely β-glucanases, indicating a potential protective role against ice-induced cellular damage [3]. Other studies also suggest that β-1,3-glucanases not only play a crucial role in plant defence against fungal pathogens but may also contribute to cold tolerance [4]. Results reported by Hon et al. (1995) [5] suggest that subtle structural modifications in PR proteins accumulating during cold acclimation in winter rye may enable them to bind ice and thus increase winter hardiness. In cold-acclimated leaves of winter rye (*Secale cereale* L.), the most abundant apoplastic proteins identified were antifreeze proteins, which show similarity to three classes of PR proteins: endochitinases, β-1,3-glucanases, and thaumatin-like proteins.

Thibaud et al., 2004 [6] indicated a potential relationship between PR protein synthesis and sucrose accumulation. Since the induction of PR-2 (β-1,3-glucanase) and PR-5 (TLP) genes in Arabidopsis thaliana leaves by sucrose or glucose cannot be attributed to osmotic effects, sucrose and/or glucose are likely to function as regulators of PR gene expression. Unlike glucose and fructose, sucrose is capable of maintaining glycolytic activity through the sucrose synthase pathway, thereby enhancing the tolerance of acclimated roots to anoxic conditions [7]. According to Ricard et al. (1998) [8], sucrose exported from the phloem, rather than hexoses, is the principal carbon source in maize root tissues under oxygen-deficient conditions. In barley, HvSUS1 and HvSUS3 were strongly induced by flooding [9] and CsSUS3-antisense cucumber plants showed reduced tolerance to flooding stress [10].

At the molecular level, flooding under low-temperature conditions activates various signalling pathways, including those involving reactive oxygen species (ROS) generation, which plays a central role in redox-dependent signalling in plants [11]. In plant tissues, glutathione is predominantly present in its reduced form (GSH), whereas only a small proportion occurs in its fully oxidized form (GSSG) [12]. GSH is an essential metabolite responsible for redox homeostasis, as the ratio of reduced to oxidised forms of ascorbate and glutathione reflects the redox state of cells [12]. In turn, ATP is involved in GSH synthesis in the regulation of rice plant cold tolerance [13]. The most fundamental and earliest function of glutathione is in the thiol–disulphide exchange, in which reduced glutathione (GSH) is continuously oxidised to the disulphide bond form (GSSG) that is reduced back to GSH by NADPH-dependent glutathione reductase (GR) [14]. Glutathione reductase (GR) is a NADPH-dependent oxidoreductase which catalyses the conversion of GSSG to GSH. Some members of the diverse glutathione transferase (GST) enzyme family have GSH-dependent thiol transferase activity and participate in the recycling of antioxidants (ascorbate, flavonoids, quinones), while other isoenzymes, due to their S-transferase activity, are involved in the detoxification mechanisms using GSH as a co-substrate [14].

Aside from maintaining cellular redox homeostasis and its oxidized form GSSG, GSH is also a key component in the ascorbate–glutathione (AsA-GSH) and glyoxalase cycles. GSH contributes to the removal of excess H_2_O_2_, a relatively stable form of ROS, and reduces methylglyoxal (MG) levels in response to environmental stresses [15]. Furthermore, GSH also participates in the modulation of gene expression that activates redox-sensitive transcription factors and enzymatic activities by regulating the redox state of plants. GSH also contributes to the detoxification of xenobiotics through glutathione S-transferase (GST)-catalysed reactions and to the sequestration of heavy metals into vacuoles through the formation of phytochelatins (PCs). GSH and the GSH/GSSG ratio act as a key redox signal in plant cells [15]. By regulating the cellular redox state, GSH indirectly may influence NONEXPRESSOR OF PATHOGENESIS-RELATED GENES 1 (*NPR1*) activity which is a central regulator of the immune response (especially the salicylic acid pathway) [16].

The 1-SST gene encodes sucrose 1-fructosyltransferase, an enzyme that catalyses the first step in fructan biosynthesis, thereby contributing to fructan accumulation during cold acclimation in cereals [17]. Fructans represent an important carbohydrate reserve in overwintering cereals, providing an energy source for the maintenance and survival of plant tissues during winter and supporting regrowth after winter stress. In turn, CBFs are transcription factors that act as key regulators of cold responses and cold acclimation [18] and are also involved in responses to other environmental stresses [19]. In the *Pooideae* subfamily, two groups of genes, *CBFIII* and *CBFIV*, are particularly important for cold acclimation [20].

To date, the interaction between cold flooding and frost tolerance remains not fully understood. Hence, the aim of this study was to investigate how flooding under low temperature conditions affected the frost tolerance of two cold-acclimated winter rye lines differing in resistance to snow mould, the occurrence of which is favoured by humid conditions. Particular attention was given to the accumulation of PR proteins, sucrose metabolism, glutathione metabolism, and expression of cold response-related genes such as *1-SST* and *CBFIV*. The analysis of glu8 and *TLP3* gene expression was intended to complement the analysis of β-1,3-glucanase isoforms at the protein level and to determine whether changes in protein accumulation were associated with corresponding changes in transcript abundance. In addition, *TLP3* expression was analysed to assess the response of another PR protein involved in plant defence under the applied stress conditions. The expression of *1-SST* and *CBFIV* was analysed to determine whether additional flooding stress modified the cold-response regulatory pathway and fructan metabolism in rye lines differing in their resistance to snow mould.

## 2. Results

### 2.1. Snow Mould Resistance

Line 343 exhibited significantly greater snow mould resistance than line 620, as reflected by the higher regrowth dry weight after inoculation (51.3% and 11.9% of the non-inoculated control, respectively) (Table 1).

### 2.2. Frost Tolerance

**Figure 1:**
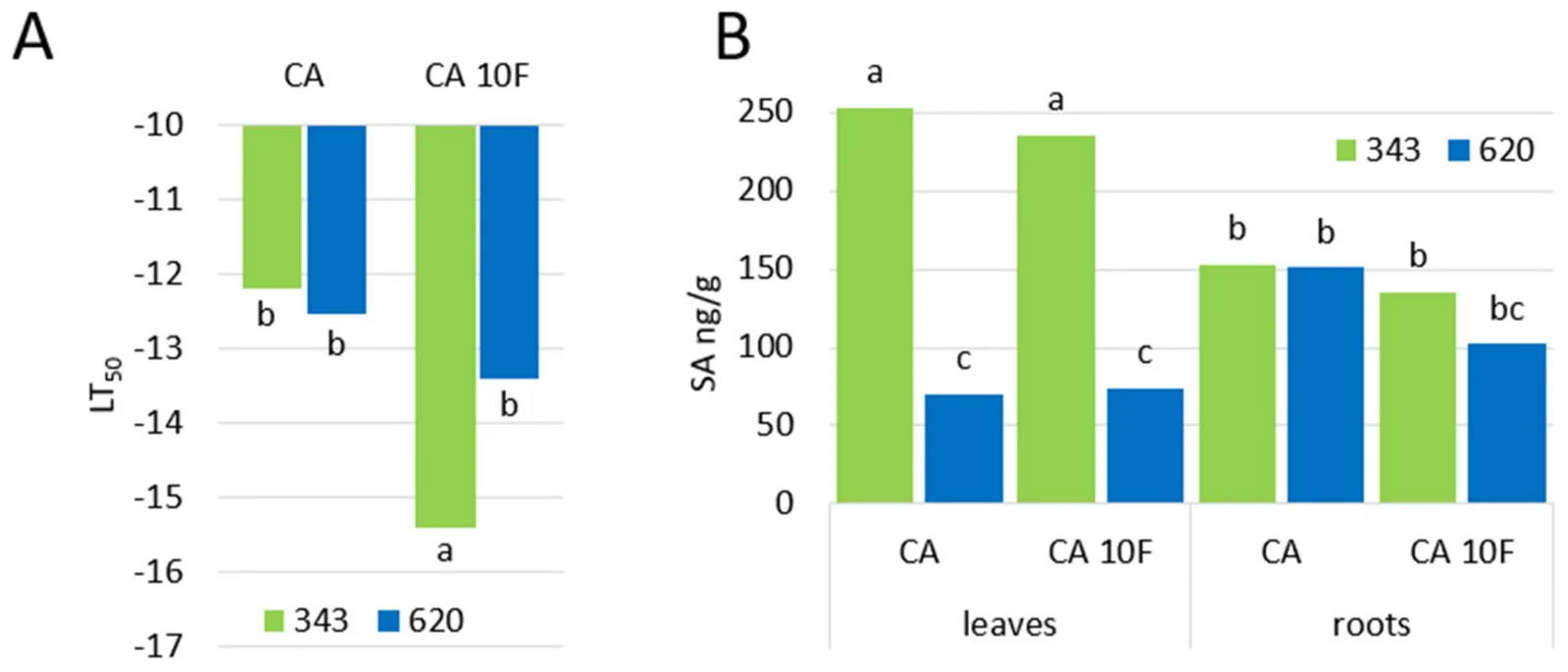
(**A**) Frost tolerance expressed as LT_50_; (**B**) Salicylic acid content in leaves of two winter rye lines (343—resistant to snow mould, 620—susceptible to snow mould), cold acclimated and then subjected to ten days (CA 10F) flooding stress under cold. Transcript levels are normalized to actin and expressed as fold change relative to control. Significant differences between treatments are indicated by different letters (*p* ≤ 0.05).

After cold acclimation alone, LT_50_ values indicated no significant difference between the two investigated lines. Following ten days of flooding treatment, the frost tolerance of line 343 was markedly enhanced (Figure 1A), suggesting that the combined stress of cold and excess of water improved frost tolerance. In contrast, line 620 showed no significant improvement.

### 2.3. Salicylic Acid

In genotype 343, salicylic acid (SA) levels were markedly higher in leaves than in roots and remained relatively stable after prolonged flooding under cold conditions (Figure 1B). In contrast, genotype 620 showed consistently lower SA content in leaves and stable levels after flooding in roots. After prolonged flooding, SA levels in roots became more similar between the two genotypes.

### 2.4. Cold Response-Related Gene Expression

**Figure 2:**
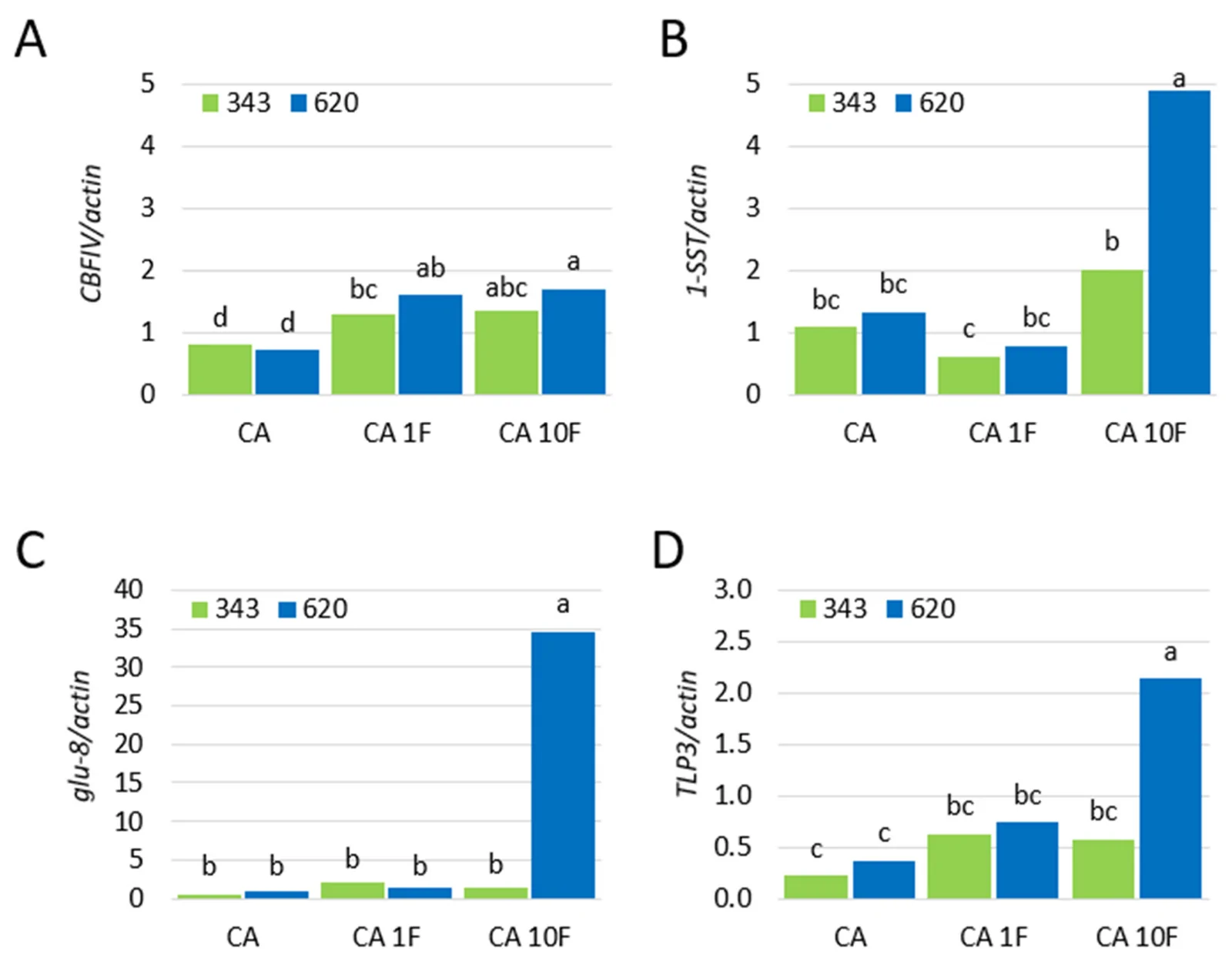
Relative transcript levels of cold-related genes: CBFIV (**A**), 1-SST (**B**), glu-8 (**C**), TLP3 (**D**) in leaves of two winter rye lines (343—resistant to snow mould, 620—susceptible to snow mould), cold acclimated and then subjected to one (CA 1F) or ten (CA 10F) days of flooding stress under cold. Transcript levels are normalized to actin and expressed as fold change relative to control. Significant differences between treatments are indicated by different letters (*p* ≤ 0.05).

In both lines, flooding caused sustained upregulation of *CBFIV* (Figure 2A) genes after one and ten days. Both lines show minimal baseline levels under CA, followed by a uniform, moderate upregulation under one and ten days of flooding. Transcript levels of the 1-SST (Figure 2B) gene remained low under cold acclimation and one day of cold flooding, followed by induction under prolonged exposure to cold flooding, but line 620 strongly outperformed the moderate increase seen in line 343. The *glu*-8 gene demonstrated completely suppressed expression across almost all treatments and remained entirely unresponsive (Figure 2C). The sole exception was a massive spike in transcript abundance observed in line 620 with ten days of flooding. Baseline expression of the TLP gene was minimal under cold acclimation, followed by a slight increment in both lines during the one-day flooding phase (Figure 2D). Prolonged cold flooding triggered a sharp, exclusive peak in line 620.

### 2.5. β-1,3-Glucanase Abundance and Activity

**Figure 3:**
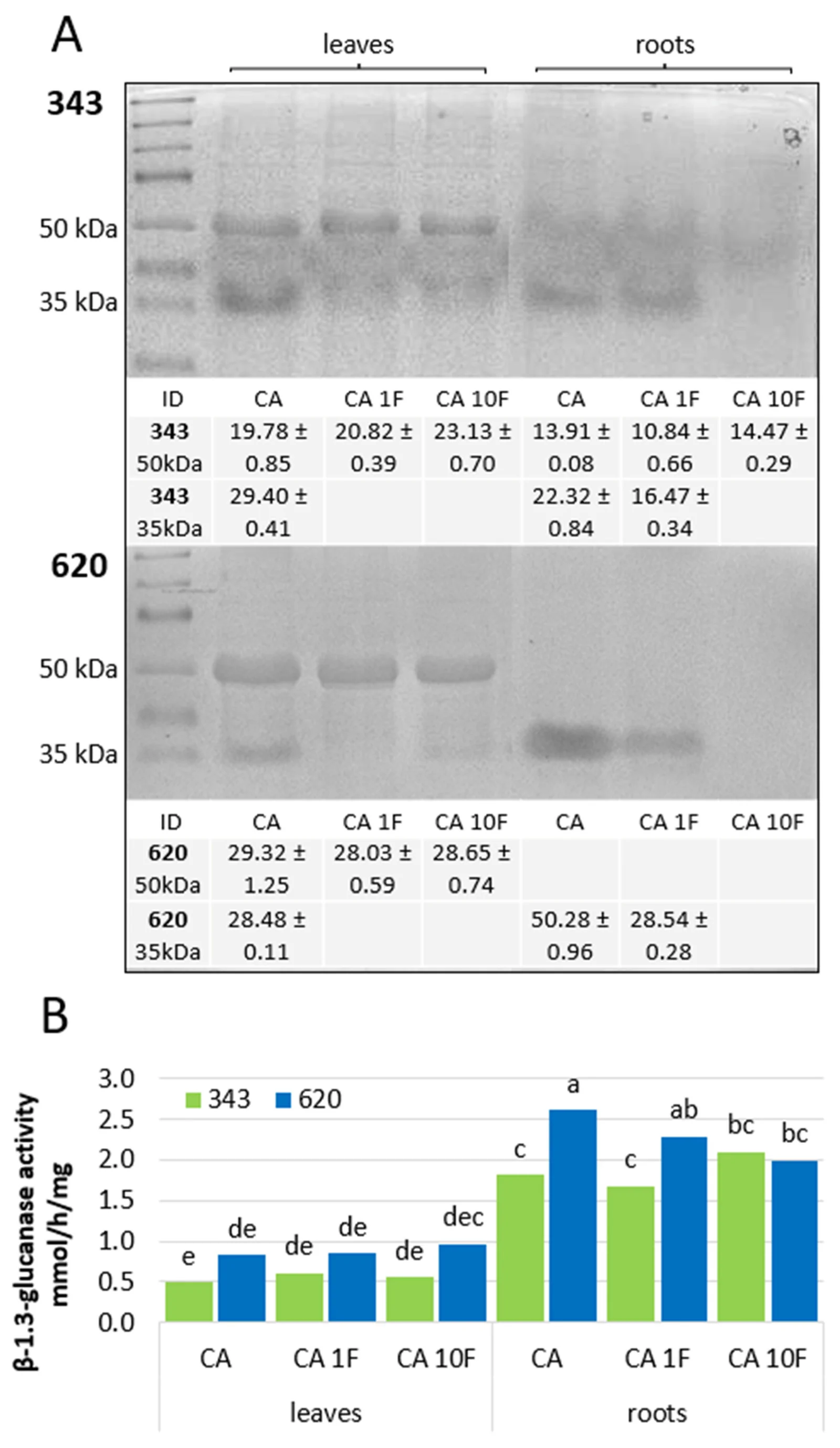
The β-1,3-glucanase isoform profile in leaves and roots detected by SDS-PAGE (**A**); Enzymatic activity of β-1,3-glucanase in leaves and roots (**B**) of two winter rye lines (343—resistant to snow mould, 620—susceptible to snow mould), cold acclimated and then subjected to one (CA 1F) or ten (CA 10F) days flooding stress under cold. Significant differences between treatments are indicated by different letters (*p* ≤ 0.05).

SDS-PAGE analysis revealed two isoforms of β-1,3-glucanase (Figure 3A). In line 343, both isoforms were consistently present in leaves and roots after flooding. In line 620, the 50 kDa isoform was not detected in the roots, while the 35 kDa isoform disappeared after ten days of flooding, resulting in the absence of both isoforms in the roots at this time point. Interestingly, lack of 50 kDa isoform was compensated by higher abundance of isoform 35 kDa in the roots of line 620.

Despite the higher β-1,3-glucanase activity observed in the roots of genotype 620 after one day of flooding (Figure 3B), the absence of the 50 kDa isoform was associated with reduced frost tolerance. The elevated enzymatic activity may reflect a compensatory response to the reduced abundance or absence of specific β-1,3-glucanase isoforms.

### 2.6. Sucrose Synthase and Sucrose Phosphate Synthase Activity

**Figure 4:**
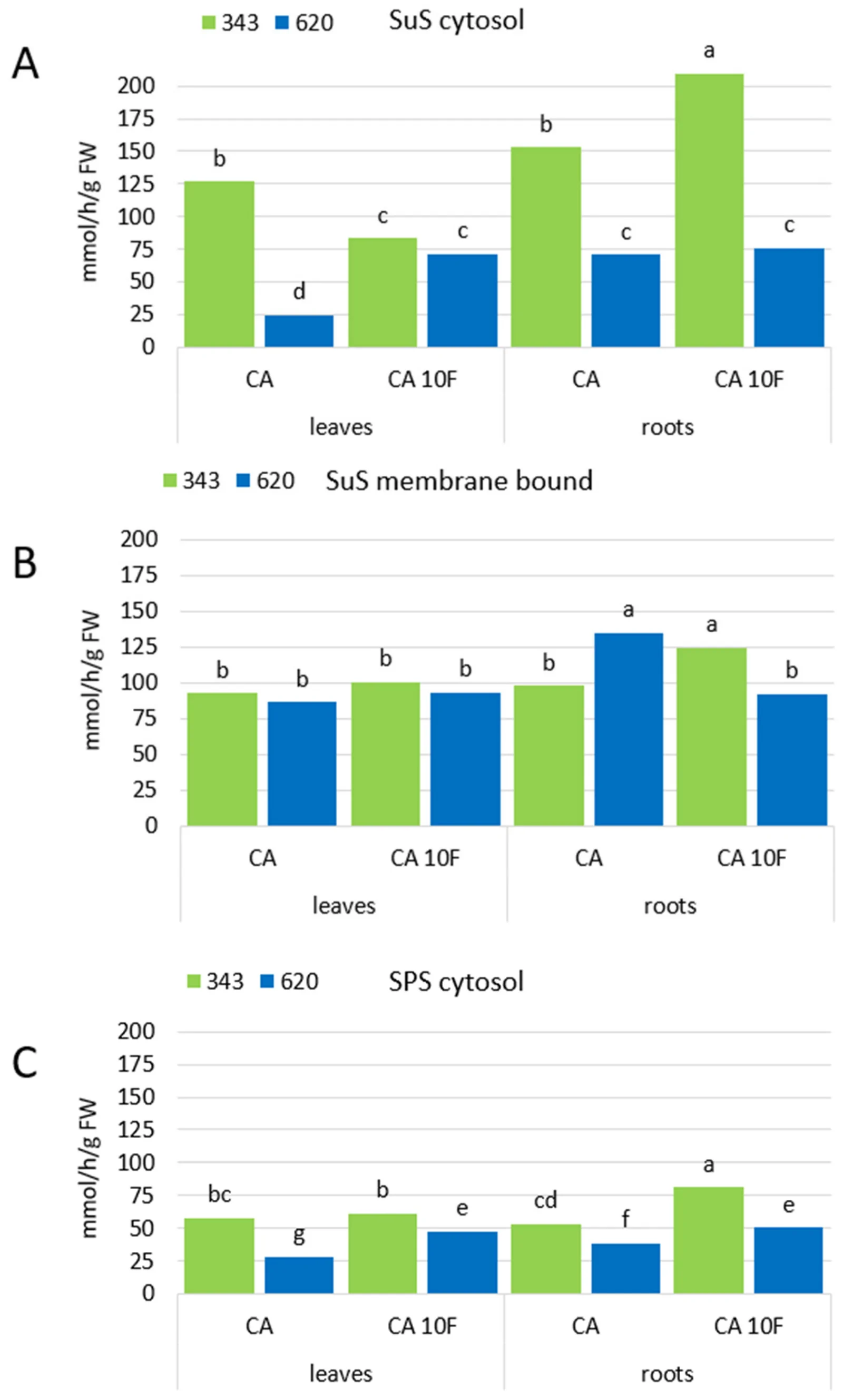
Activity of cytosolic (**A**) and membrane-bound (**B**) sucrose synthase (SuS); activity of sucrose phosphate synthase (SPS) (**C**) in leaves and roots of two winter rye lines (343—resistant to snow mould, 620—susceptible to snow mould), cold acclimated and then subjected to ten (CA 10F) days flooding stress under cold. Significant differences between treatments are indicated by different letters (*p* ≤ 0.05).

Sucrose synthase (SuS) activity was measured in both cytosolic and membrane-associated fractions. The two genotypes showed different responses in the cytosolic fraction. In line 343, flooding decreased SuS activity in leaves, while root activity increased. Conversely, in line 620, cytosolic SuS activity in leaves increased significantly under flooding whereas activity in roots remained stable even after 10 days of flooding (Figure 4A). In the membrane fraction, flooding caused no significant changes in SuS activity in the leaves of either genotype. In contrast, in the roots, SuS activity increased in line 343 but decreased in line 620 following flooding (Figure 4B).

Sucrose Phosphate Synthase (SPS) activity in the cytosol showed that line 343 consistently maintained significantly higher levels than line 620 across all conditions. In line 343, significant increase after flooding were observed only in roots. In both leaves and roots, the transition from cold acclimation to prolonged flooding (10 days) under low-temperature conditions resulted in increased activity in line 620 (Figure 4C).

### 2.7. Glutathione Metabolism

Glutathione level showed distinct tissue-specific patterns. Across both lines and treatments, the highest concentrations were observed in leaves, while roots consistently exhibited the much lower level. In leaves of both genotypes, GSH and total glutathione levels increased progressively under CA 1F and CA 10F conditions, indicating strong activation of the antioxidant system during prolonged stress.

In roots of both genotypes, total glutathione content increased markedly under CA 1F treatment but decreased significantly under CA 10F. GSSG represented a relatively larger proportion of total glutathione pool, especially under cold acclimation, which may indicate a more oxidized cellular environment. This pattern suggests elevated levels of ROS and a less efficient GSH regeneration, as GSH is oxidized to GSSG while detoxifying ROS under stress conditions. Therefore, a higher proportion of GSSG relative to GSH indicates that more glutathione is being consumed in antioxidant reactions and that the cellular redox state becomes more oxidized. In line 620, total glutathione decreased in roots after 10 days of flooding, coinciding with loss of the 50 kDa β-1,3-glucanase isoform and reduced frost tolerance. These results highlight the importance of antioxidant activity in roots, the organs directly exposed to anoxic stress.

**Figure 5:**
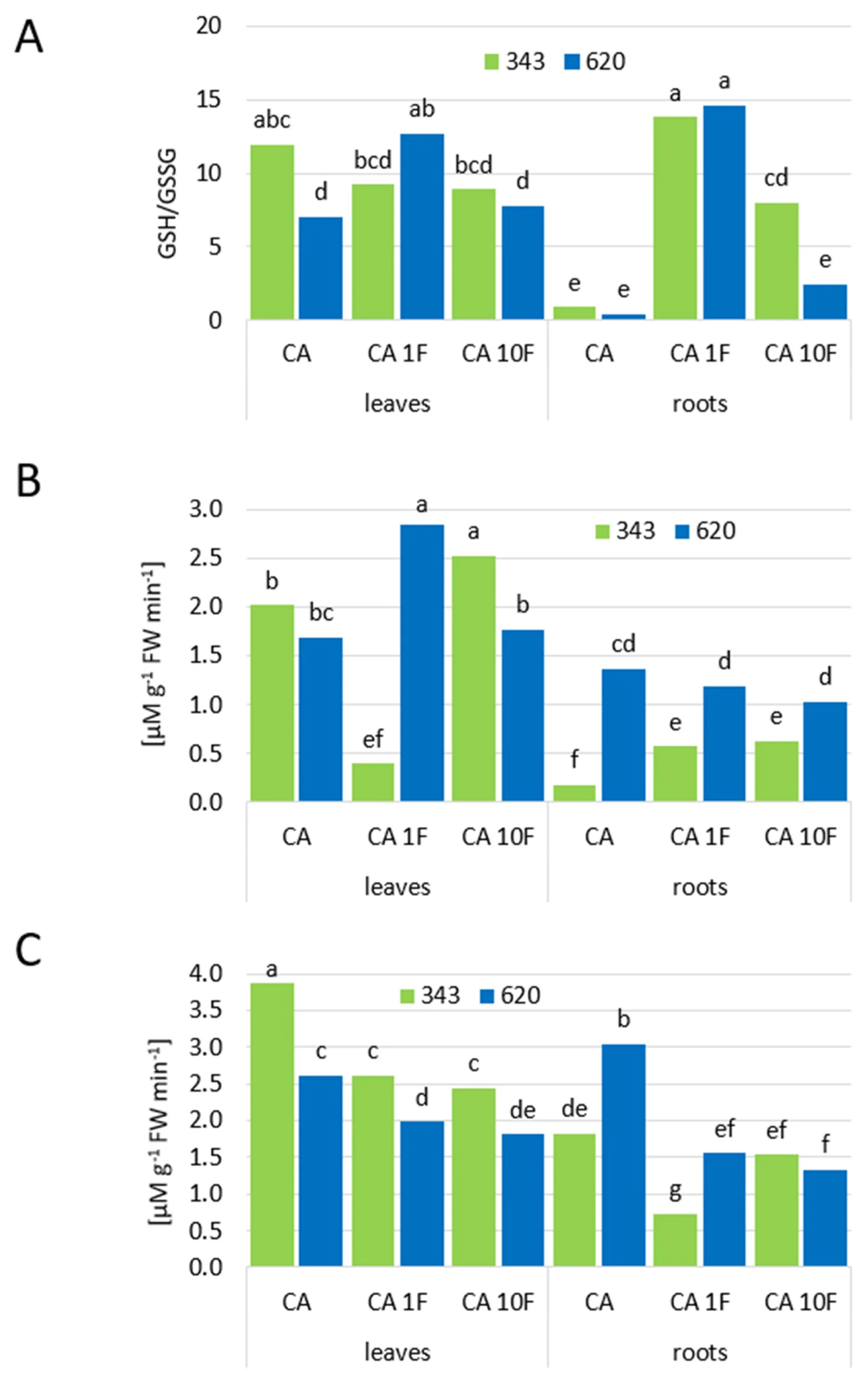
Reduced (GSH) and oxidised (GSSG) glutathione ratio (**A**), glutathione reductase (GR) activity (**B**); glutathione transferase (GST) activity (**C**) in leaves and roots of two winter rye lines (343—resistant to snow mould, 620—susceptible to snow mould), cold acclimated and then subjected to one (CA 1F) or ten (CA 10F) days stress under cold. Significant differences between treatments are indicated by different letters (*p* ≤ 0.05).

Leaves of genotype 343 under CA 1F conditions maintained high GSH levels (Table 2) despite the pronounced decrease in GR activity (Figure 5B) and a significant reduction in GST activity (Figure 4C). In both genotypes, CA 1F treatment resulted in substantially higher GSH content compared with CA alone, indicating that flooding strongly stimulated accumulation of reduced glutathione. In genotype 620, GST activity also decreased significantly relative to CA and remained markedly lower than in genotype 343 (Figure 5C), demonstrating weaker GSH-dependent detoxification capacity. Accordingly, the GSH/GSSG ratio (Figure 4A) increased significantly under CA 1F only in genotype 620, whereas genotype 343 showed no substantial change, indicating that the sensitive line showed a stronger shift toward a more reduced redox state despite its lower detoxification capacity. Despite the substantial suppression of GST under CA 1F, genotype 343 was more effective in maintaining glutathione homeostasis, sustaining a high GSH pool despite reduced GR- and GST-dependent turnover. This ability of genotype 343 to preserve high GSH levels is likely to reflect efficient detoxification and controlled GSH consumption without excessive oxidation of the glutathione pool. Also, prolonged stress (CA 10F) did not induce substantial glutathione oxidation in this genotype, allowing the maintenance of the GSH pool even in the absence of strong enzymatic recycling. Thus, in genotype 343, GSH stability during flooding appears to be associated mainly with balanced detoxification rather than with GR activation. Under CA 10F, the further increase in leaf GSH levels was probably supported by the substantial increase in GR activity, while GST activity remained at a moderate level. In contrast, genotype 620 exhibited a pronounced increase in GR activity under CA 1F, indicating a stronger reliance on GR-mediated recycling of oxidized glutathione to sustain the reduced GSH pool. However, because GSH levels remained comparatively low, the GR-dependent regeneration was insufficient to compensate for GSH consumption and oxidation, resulting in a less effective redox response.

In the roots, GR activity showed a clearly divergent response between the two genotypes. In genotype 343, GR activity increased significantly under both CA 1F and CA 10F treatments, indicating an enhanced capacity for enzymatic regeneration of reduced glutathione. In contrast, the sensitive genotype 620 exhibited no substantial changes in GR activity across treatments, suggesting a limited ability to adjust GR-dependent antioxidant defense under stress. GST activity also differed between the genotypes. In genotype 343, GST activity increased significantly under CA 1F and remained at a comparable level under CA 10F, indicating sustained GSH-dependent detoxification capacity. In genotype 620, GST activity did not change significantly relative to CA, further demonstrating the limited responsiveness of the detoxification system in the roots of the sensitive line (Figure 5B,C). Consistent with these enzymatic patterns, the GSH/GSSG ratio (Figure 5A) increased significantly under CA 1F in both genotypes, but under prolonged stress (CA 10F), a further increase was observed only in genotype 343, and this rise was smaller than that recorded after CA 1F, indicating a moderate but sustained redox-stabilizing response in the tolerant line.

### 2.8. PCA of Responses to Flooding Under Low-Temperature Conditions

**Figure 6:**
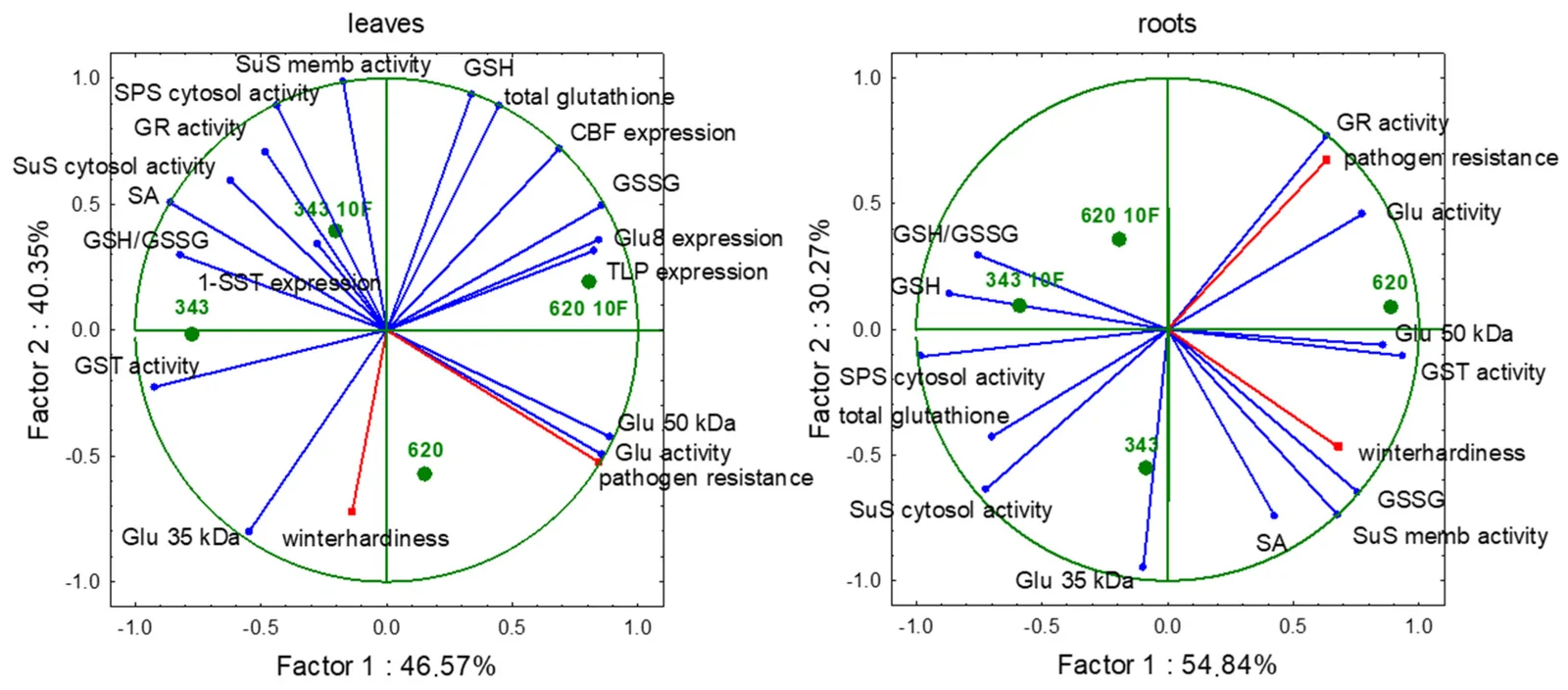
The first two principal components (PC1 and PC2) in leaves and roots of cold-acclimated control plants and cold-acclimated plants following ten days of flooding (CA 10F). Red vectors indicate supplementary variables (winterhardiness and pathogen resistance) used to assess their relationships with the analysed parameters.

PCA analyse revealed distinct physiological response patterns in leaves and roots under flooding stress (Figure 6). The first principal component (Factor 1) primarily captured the variation associated with flooding stress, whereas the second principal component (Factor 2) reflected genotype differences independent of stress. In the leaf tissue, these two components contributed nearly equally the total variance, with Factor 1 explaining 46.57% and Factor 2 explaining 40.35% (86.92% cumulative). In contrast, the root system was dominated by the primary stress-response axis (Factor 1), which accounted for 54.84% of the total variability, while genotype differences (Factor 2) explained 30.27%, resulting in a cumulative variance of 85.11%. This divergence indicates that flooding stress exerts a stronger and more uniform influence on root physiology, whereas leaf responses integrate both stress-dependent and genotype-specific components.

The control leaves of genotype 343 were associated with GST activity, the GSH/GSSG ratio, salicylic acid (SA), GR activity, and cytosolic SPS activity, indicating efficient redox regulation and active carbohydrate metabolism. In contrast, the control leaves of genotype 620 clustered with pathogen resistance traits, including β-1,3-glucanase activity, the 50 kDa Glu protein, TLP expression, Glu8 expression, and GSSG accumulation, forming a defence-oriented response pattern. Following 10 days of flooding, leaves of genotype 343 were associated primarily with membrane-bound and cytosolic sucrose synthase activities, cytosolic SPS activity, 1-SST expression, and elevated SA levels. This pattern suggests substantial metabolic reprogramming in the tolerant genotype, particularly through the activation of carbohydrate metabolism and stress-signalling pathways. In contrast, flooded leaves of genotype 620 remained associated with pathogen resistance-related traits, including β-1,3-glucanase activity, the 50 kDa Glu protein, TLP expression, and Glu8 expression. Their position on the positive side of the PCA plot further suggests an enhanced contribution of defence-related mechanisms under flooding conditions. Overall, the PCA highlights contrasting strategies in response to flooding; genotype 343 was characterized mainly by metabolic adjustment, whereas genotype 620 showed a stronger association with defence-related and stress-responsive traits.

The control roots of the 343 genotype were associated with cytosolic SuS activity, cytosolic SPS activity, and, to a lesser extent, total glutathione which indicates a close relationship with carbohydrate metabolism. It was also positioned opposite to the cluster formed by pathogen resistance, β-1,3-glucanase activity, GST activity, and the 50 kDa Glu protein. The control roots of the 620 genotype were closely associated with pathogen resistance, β-1,3-glucanase activity (Glu activity), GST activity, and the 50 kDa Glu protein. Following 10 days of flooding, roots of 343 genotype were most closely associated with GSH content and the GSH/GSSG ratio, indicating activation of glutathione-dependent redox regulation in response to flooding. Compared with the control plants, flooding therefore altered the profile of the resistant genotype toward glutathione metabolism. The flooded sample of roots of the 620 genotype showed the strongest association with GR activity, pathogen resistance, β-1,3-glucanase activity, and the presence 50 kDa Glu protein.

Overall, in leaves, the response to environmental stimuli was closely linked to genotype-specific traits. Control 620 genotype was strongly associated with vectors representing pathogen-related defence traits including PR proteins. In contrast, the 343 control genotype appeared to avoid the typical defence response reactions and was linked to glutathione and sucrose synthesis enzyme activity. The vector labelled “winterhardiness” represents LT_50_ values, where lower (more negative) values indicate greater freezing tolerance. After flooding, the genotype 343 was located on the opposite side of the LT_50_ vector and therefore appears to be associated with higher freezing tolerance. In contrast, genotype 620, positioned closer to the LT_50_ vector, was associated with higher LT_50_ values and consequently lower freezing tolerance. In roots, the PCA biplot showed a close alignment of the Glu 50 kDa vector with pathogen resistance, β-1,3-glucanase activity (Glu activity), and GST activity.

## 3. Discussion

This study provides new insights into how root flooding during cold acclimation modifies frost tolerance in winter rye lines differing in resistance to the pathogen causing snow mould. The two winter rye lines did not differ in frost tolerance after cold acclimation, although they differed in their resistance to *Microdochium nivale*. Differences in frost tolerance between the lines emerged only after exposure to cold flooding. Flooding enhanced frost tolerance in the snow mould-resistant line 343, whereas no such effect was observed in the susceptible line 620. Increased frost tolerance was mainly associated with organ-specific β-1,3-glucanase isoform accumulation and non-enzymatic antioxidant defence capacity, consistent with earlier observations that β-1,3-glucanase isoforms contribute to stress-response and pathogen-tolerance mechanisms in winter triticale [21]. Short-term flooding (CA 1F) induced a weaker response in both rye lines, whereas prolonged flooding (CA 10F) resulted in a strong divergence between the genotypes. The initial stress perception, including early transcriptional activation of cold-responsive genes, was similar in both lines, whereas differences in frost tolerance arose from distinct metabolic adaptations developing during prolonged flooding.

Expression of PR-proteins genes is strongly regulated by salicylic acid-mediated pathways activated during oxidative stress [16]. Previous studies have demonstrated that waterlogging can induce SA changes in different way in roots and basal stem nodes [22]. The lack of genotypic differences in SA content in roots, despite the pronounced differences observed in leaves, may reflect tissue-specific regulation of SA metabolism under prolonged flooding. Root flooding triggers hypoxia signalling by activating plasma membrane Ca^2+^ channels, thereby initiating a systemic alert throughout the plant and inducing acclimation responses in distant tissues [23]. In this study, roots were directly exposed to flooding and may therefore have developed robust responses to stress, which could explain the lack of differences between the genotypes. In contrast, the higher SA content maintained in the leaves of the resistant genotype may reflect differences in the systemic or shoot-specific regulation of stress and defence responses.

PR proteins, including β-1,3-glucanase (glu-8) and thaumatin-like proteins (TLP), are commonly regarded as markers of both biotic and abiotic stress responses [4]. Their transcriptional activation may reflect defence responses associated with progressive oxidative damage and persistent redox imbalance, as indicated by the increased glutathione pool observed under prolonged flooding conditions. In the present study, SDS-PAGE analysis demonstrated that β-1,3-glucanase occurs in two main isoforms, whose presence and abundance depend on genotype and flooding stress. In line 343, both isoforms remained detectable in leaves and roots even after flooding, suggesting a sustained response to prolonged stress. In line 620, the 50 kDa isoform was absent from the roots after prolonged flooding, whereas the abundance of the 35 kDa isoform increased. Although β-1,3-glucanase activity was high after one day of flooding, the loss of the 50 kDa isoform may have been associated with the lower frost tolerance observed in line 620. The increased glu-8 transcript abundance observed in the susceptible line after 10 days of flooding was not accompanied by the accumulation of the 50 kDa glucanase isoform or by increased β-1,3-glucanase activity. This discrepancy suggests that glu-8 expression may be subject to post-transcriptional regulation, as similar discrepancies between mRNA abundance and β-1,3-glucanase protein accumulation have previously been reported [24,25]. These findings suggest that not β-1,3-glucanase activity overall, but rather, the presence of specific isoforms, may be important for protecting plants against freezing stress. β-1,3-Glucanases are known to participate in cell wall reinforcement during plant defence against pathogens. However, specific isoforms associated with the maintenance of cell wall structural integrity may also contribute to tolerance to abiotic stresses, including flooding/submergence, oxidative stress, and freezing. This interpretation is further supported by the considerable diversity of PR proteins. According to Sock et al. (1990) [26], wheat leaves contain multiple constitutive endo-β-1,3-glucanase isoforms, highlighting the high molecular diversity of these enzymes even in non-stressed tissues. Different experimental approaches have shown that apoplastic antifreeze polypeptides from cold-acclimated winter rye are closely related to pathogenesis-related (PR) proteins, including β-1,3-glucanases, endochitinases, thaumatin-like proteins, and lipid transfer proteins [5]. These proteins are involved in controlling ice crystal growth in the apoplast [27] and may occur in complexes containing β-1,3-glucanase isoforms of approximately 32 and 34 kDa [28].

Glutathione metabolism is closely linked to both freezing tolerance and resistance to fungal pathogens, as glutathione is a central regulator of cellular redox homeostasis under stress conditions. During cold stress, enhanced glutathione synthesis and recycling protect membranes and proteins against oxidative damage caused by ROS, thereby improving frost tolerance and maintaining metabolic stability [29]. Flooding-induced oxidative stress was reflected in organ-specific differences in glutathione levels. Leaves, which contained the highest glutathione levels, appeared to be less affected, whereas roots consistently had the lowest antioxidant pools. In line 620, glutathione content in the roots decreased significantly after prolonged flooding, coinciding with the loss of the 50 kDa glucanase isoform and reduced frost tolerance. In contrast, line 343 maintained relatively stable glutathione levels across both organs, suggesting more effective protection against oxidative damage. These results are consistent with earlier reports indicating that antioxidant capacity under flooding is an important determinant of stress tolerance, particularly in underground tissues directly exposed to water excess. The accumulation of GSH in line 343 under prolonged flooding may reflect sustained activation of antioxidant and detoxification mechanisms that help prevent excessive oxidation of the glutathione pool. In contrast, the lower GSH content, reduced GSH/GSSG ratio, and lower salicylic acid (SA) level in line 620 may be associated with altered redox-dependent signalling and potentially weaker coordination of downstream defence responses under flooding. Since SA accumulation is involved in NPR1-dependent regulation of PR-mediated defence responses, reduced SA content may have contributed to the lower accumulation of PR protein isoforms observed in the roots. Increased glutathione concentrations have been reported to promote SA accumulation and activation of NPR1-dependent defence responses [23]. NPR1 acts as a redox sensor, undergoing redox-dependent changes in its oligomeric state that regulate downstream immune responses [16,30,31]. Increased GSH levels and SA accumulation, for example, during pathogen attack, can shift the cellular redox state towards a more reducing environment, which promotes NPR1 monomerization from disulfide-linked oligomers, its translocation to the nucleus, and subsequent activation of PR genes [30,31]. Although NPR1 monomerization was not directly assessed in the present study, the lower GSH and SA levels observed in line 620 may indicate less favourable redox and signalling conditions for NPR1-dependent defence responses. This possibility may contribute to explaining the absence of the 50 kDa β-1,3-glucanase isoform in the roots of genotype 620 under flooding conditions.

The metabolism of low-molecular-weight carbohydrates has previously been identified as a factor linking frost tolerance and resistance to snow mould in *Festulolium*, with genotypes exhibiting higher photosynthetic efficiency accumulating greater amounts of soluble carbohydrates and consequently showing enhanced resistance to *Microdochium nivale* as well as improved freezing tolerance [32]. Sucrose is not only a substrate for the 1-SST enzyme but also an important component of plant stress tolerance, contributing to both pathogen defence and frost resistance. It serves as an energy source and signalling molecule during defence responses against pathogens.

During prolonged flooding, the susceptible line 620 showed a strong increase in 1-SST expression, whereas line 343 maintained a more stable transcript level. The higher cytosolic SuS and SPS activities maintained in the leaves of line 620 after 10 days of flooding could support continued sucrose production, thereby providing substrate for fructan synthesis and sustaining carbohydrate metabolism under flooding stress. At the same time, decreased cytosolic SuS activity in line 343 may restrict sucrose biosynthesis in leaves, although increased SuS activity was observed in the roots. The higher cytosolic SuS activity observed in the roots of line 343 may indicate a greater capacity to maintain sucrose turnover and carbohydrate homeostasis under flooding stress, potentially supporting the regulation of soluble sugar pools, osmotic balance, and carbon transport in this organ. In contrast, the lower SuS activity observed in the roots of line 620 may suggest restricted carbon turnover and disturbed carbon allocation within root tissues. The lack of significant differences in the membrane-bound SuS fraction activity, particularly in the leaves of flooded plants, may indicate that membrane-bound SuS activity is more stable and less responsive to flooding stress than the cytosolic fraction. In contrast, in the roots, flooding increased membrane-bound SuS activity in line 343, whereas it decreased in line 620. Membrane-associated SuS is often linked to structural and maintenance functions, such as cell wall metabolism, which may enable the maintenance of basic cellular integrity and root functionality during flooding. Under control conditions, Albrecht et al. (1993, 1997) [33,34] did not observe major differences in sucrose distribution along the root axis, whereas under hypoxia, sucrose levels increased at least twofold in roots. The flooding-induced activation of SuS in the roots of line 343 may be particularly relevant under low-temperature conditions, as sucrose acts as a compatible solute during cold stress, stabilizing cellular membranes and proteins and protecting cells against dehydration, thereby potentially contributing to the maintenance or enhancement of frost tolerance.

## 4. Material and Methods

### 4.1. Plant Material and Growth Conditions

Two inbred winter rye (*Secale cereale* L.) lines originating from the collection developed at Plant Breeding and Acclimatization Institute (IHAR), Radzików, Poland, were used. Based on four-year field evaluations of snow mould resistance, line 343 was shown to be significantly more resistant to *Microdochium nivale* than line 620 [35]. The experiments were arranged in a completely randomized design. The seeds were sown in pots (16 plants per 25-cm-diameter pot) containing a mixture of soil and sand (2:1, *v*/*v*) and grown for 3 weeks in a greenhouse at 18 °C (day/night) under natural daylight conditions. The experiment was conducted in October in Kraków, Poland (50°03′ N, 19°55′ E). At the three-leaf stage, the plants were cold-acclimated in a growth chamber for 3 weeks at 4 °C under an 8 h photoperiod with a light intensity of 200 µmol m^−2^ s^−1^ photosynthetic photon flux density (PPFD), using AGRO Philips sodium lamps. After 3 weeks, the plants were divided into two groups. One group remaining under the same cold-chamber conditions was subjected to flooding by placing the pots up to their rims in a container filled with tap water for 10 days. Samples were collected separately from leaves and roots directly after the 3-week cold-acclimation period (CA) and after 1 (CA 1F) and 10 days of flooding (CA 10F). The second group was used to assess resistance to *Microdochium nivale*.

### 4.2. Microdochium Nivale Inoculation and Evaluation of Regrowth

Resistance to *Microdochium nivale* was evaluated in cold-acclimated plants. Half of the plants were inoculated with *M. nivale* mycelium (isolate No. 38z/5a/01), collected from winter rye in 2001 and provided by Prof. Maria Prończuk from the Plant Breeding and Acclimatization Institute in Radzików, Poland, while the other half served as non-inoculated control. The inoculum preparation and inoculation were performed as described by Żur et al. (2011) [36] and Pociecha et al. (2013) [37]. Briefly, the inoculum was prepared by growing the fungus in flasks containing soil medium at 18–20 °C in darkness for 7 days. After colonization of the soil medium by the mycelium, the macerated medium was added to the soil of pots with plants dedicated for inoculation. The inoculated and non-inoculated plants were covered with moistened blotting paper and plastic foil to maintain high humidity and incubated at 4 °C in darkness for disease development. After 30 days, the blotting paper and plastic foil were removed, and the plants were cut and allowed to regrow for 3 days at 12 °C, followed by 7 days at 18 °C (8-h photoperiod, 200 µmol m^−2^ s^−1^ PPFD). The initial temperature of 12 °C was used to avoid thermal shock following cold incubation, while the subsequent increase to 18 °C provided conditions for regrowth, which is strongly limited at low temperatures. After this period, the regrowth was harvested and dried to constant mass. The results were expressed as a percentage of the regrowth dry biomass of the non-inoculated control plants.

### 4.3. Frost Tolerance

LT_50_ was estimated on whole leaves using electrolyte leakage tests according to method described by Flint et al. (1967) [38] and Rapacz (1999) [39]. Approximately 2 cm length sections were cut from the middle part of young, fully expanded leaves and placed on ice in plastic containers. The containers were placed in a cold chamber and the temperature was lowered at a rate of 0.2 K min^−1^ until the established test temperatures (–5, –9, –15 °C) were reached, where they were held for 1 h. The temperature was then raised at the same rate until +2 °C was reached and left to thaw on a laboratory shaker for 24 h. LT_50_ was estimated using linear regression fitted to the linear relationship between temperature and cell damage, using at least three temperatures. LT_50_ measurements were performed in ten replicates for each treatment and cultivar.

### 4.4. Salicylic Acid Determination

Salicylic acid extraction and purification followed the protocol of Dziurka et al. (2016) [40]. Analyses were carried out in three independent replicates on an Agilent Infinity 1260 UHPLC apparatus (Agilent, Waldbronn, Germany), attached to a triple quadruple mass spectrometer (6410 Triple Quad LC/MS, Agilent, Santa Clara, CA, USA) and equipped with electrospray ionisation (ESI), in accordance with Sadura et al. (2019) [41].

### 4.5. β-1,3-Glucanase Protein Accumulation and Activity

β-1,3-glucanase protein accumulation was determined by SDS-PAGE as described previously [21,42]. Crude protein extracts (30 µg) were isolated from leaves and roots using an extraction buffer containing 0.1 M sodium acetate (pH 5.0) and 0.02% (*v*/*v*) β-mercaptoethanol. Protein samples were immediately frozen in liquid nitrogen and homogenized, and all subsequent extraction steps were performed under cold conditions on ice to minimize protein degradation. Protein concentration was determined spectrophotometrically in accordance with Bradford (1976) [43]. Protein extracts (10 µg) were loaded onto the gels without prior heat denaturation and separated on 12.5% (*w*/*v*) SDS-containing polyacrylamide slab gels [44], supplemented with 2.5% (*w*/*v*) laminarin from *Laminaria digitata* (Sigma L-9634), as an enzyme substrate. Electrophoresis was performed at 8 °C at a constant voltage of 120 V for 2 h. After electrophoresis, proteins were renaturated by incubating the gel in 50 mM sodium acetate buffer (pH 5.0) containing 1% (*v*/*v*) Triton X-100 for 1 h constant shaking. The β-l,3-glucanase isoforms were visualised by boiling the gel for 10 min in 1 M NaOH containing 0.1% (*w*/*v*) 2,3,5-triphenyltetrazolium chloride according to the method of Pan et al. (1991) [45]. After enzyme activity detection, the gels were stained with Coomassie Brilliant Blue R 250 for total protein profile detection.

Total β-l,3-glucanase enzymatic activities were assayed spectrophotometrically with laminarin as a substrate (Sigma L-9634), using the dinitrosalicylic acid (DNS) method [46]. For each assay, 100 µL of crude plant protein extract was used for the determination of enzyme activity. The absorbance was measured at 500 nm (Ultraspec 100, Pharmacia Biotech, Cambridge, UK). The enzyme activity was expressed in nmol as an amount of released reducing sugar (D-glucose) per hour per milligram of soluble protein. All data are the means of three replications.

### 4.6. Analysis of Transcripts Levels of Glu-8, 1-SST, TLP3 and CBFIV Genes

Quantitative relative real-time PCR analysis of *glu*-8, *1-SST*, *TLP*3 and *CBFIV* genes was conducted using QuantStudio 3 (Thermo Fisher Scientific, Watham, MA, USA). Actin was used as an endogenous control gene [47]. RNA extractions were performed using an Rneasy Plant Mini Kit (Qiagen, Hilden, Germany), and genomic DNA elimination and reverse transcription reactions were performed using a QuantiTect Reverse Transcription Kit (Qiagen) as described by Jurczyk et al. (2021) [35,48]. PCR primers and probes (Table 3) were designed using Primer Express v. 3.0.1 software (Applied Biosystems by Life Technologies, Foster City, CA, USA). PCR amplification used the protocol as described by Jurczyk et al. (2021) [48]. Data analysis was conducted using QuantStudio Design and Analysis v. 1.5.0 software for the QuantStudio 3.

### 4.7. Sucrose Synthase and Sucrose Phosphate Activity

Homogenisation and isolation of fractions (cytosolic and membranes) for sucrose synthase (SS) analyses were performed based on a modified protocol by Sommarin et al. (1985) [49] and Janeczko et al. (2008, 2013) [50,51]. Briefly, leaf or root samples (about 30 g of fresh weight) were homogenised) in 400 mL of 10 mM Tris/HCl buffer (pH 7.8) containing 0.25 M sucrose, 1 mM EDTA, 2.5 mM DTT and 10 mM sodium molybdate (Sigma-Aldrich, Poznań, Poland). The crude extracts were filtered through two layers of interfacing material and centrifuged for 10 min at 10,000× *g* (Beckmann L3-50, rotor 15, Palo Alto, CA,, USA) to remove remaining plant tissue residues including chloroplast, nuclear, and mitochondrial fractions. The resulting supernatant were then centrifuged for 30 min at 80,000× *g* (Beckmann L8-M, rotor SW 27, Palo Alto, CA, USA). The obtained pellet was considered the membrane fraction, also called the microsomal fraction. The obtained supernatant was considered the cytosolic fraction. The pellet was resuspended in the buffer described above and taken for analysis. In order to release the protein from the membranes, Triton X-100 was added. Cytosolic and membrane-bound sucrose synthase activities were measured according to Kalt-Torres and Huber’s method (1987) [52]. SuS activity was measured at 33 °C by quantifying sucrose formation using the resorcinol method. The assay mixture (350 µL) contained 215 µL of measuring buffer (7.0 µmol fructose and 3.5 µmol UDP-glucose dissolved in 50 mM HEPES-NaOH buffer, pH 7.5, containing 15 mM MgCl_2_) and 135 µL of extract. For SPS activity measurements, the measuring buffer contained 3.5 µmol fructose-6-phosphate and 8.75 µmol UDP-glucose. The reactions were terminated after 20 min by removing a 70 µL aliquot of the reaction mixture and transferring it to a tube containing 70 µL of 1 N NaOH. The tubes were boiled for 10 min to destroy any remaining fructose, followed by the addition of 250 µL of 0.1% (*w*/*v*) resorcinol in 95% ethanol and 750 µL of 30% (*v*/*v*) HCl. The tubes were incubated at 80 °C for 8 min and, after cooling, the absorbance at 520 nm (A_520_) was measured. Sucrose formation was quantified by comparison with a sucrose standard curve.

### 4.8. Extraction and Quantification of Glutathione and Glutathione Enzymes

Extraction and determination of reduced and oxidized forms of glutathione (GSH and GSSG, respectively) were determined separately for leaves and roots, as described in Gruszka et al. (2018) [53]. The total glutathione content was quantified using an enzymatic recycling assay that detected both reduced (GSH) and oxidized (GSSG) glutathione, following the protocols described by Griffith (1980) [54] and Smith (1985) [55]. Reduced GSH was calculated from the difference between total glutathione and GSSG concentrations. The cellular redox state was assessed using the GSH/GSSG ratio, calculated as the concentration of the reduced glutathione (GSH) divided by that of oxidized glutathione (GSSG).

For enzyme extraction and activity assays, leaf and root tissues were homogenized at 0–4 °C in 6 volumes of Tris–HCl buffer (50 mM, pH 7.8) containing 2 mM EDTA-Na2, 2% (*w*/*v*) polyvinylpyrrolidone K25 and 0.5 mM protease inhibitor (Pefablock, Sigma-Aldrich). Homogenates were centrifuged (12,000× *g*, 15 min, 4 °C), and the total soluble enzyme activities were measured spectrophotometrically in the supernatant. All measurements were carried out at 25 °C using an Ultraspec 2100 pro UV/Visible spectrophotometer (Amersham Biosciences, Cambridge, UK). Enzyme activities were expressed relative to fresh weight [FW] of tissue.

Glutathione reductase (GR) activity was assayed by monitoring NADPH oxidation at 340 nm with the extinction coefficient of 6.22 mM^−1^ cm^−1^ in accordance with Klapheck et al. (1990) [56]. The assay mixture consisted of 600 µL of 50 mM Tris-HCl buffer (pH 7.8), 0.1 mM NADPH, 0.6 mM GSSG, and 25 µL of enzyme extract. GR activity was expressed in µM g^−1^ FW min^−1^.

Glutathione S-transferase (GST) activity was determined by measuring the formation of the conjugate reaction product at 340 nm using 1-chloro-2,4-dinitrobenzene (CDNB) as substrate using an extinction coefficient of 9.6 mM^−1^ cm^−1^ in accordance with Mauch and Dudler (1993) [57]. The reaction mixture contained 600 µL of 0.1 M sodium phosphate buffer, pH 6.5, 1 mM EDTA-Na_2_, 0.67 mM CDNB, 4.1 mM GSH, and 25 µL of enzyme extract. The amount of GST activity was expressed as µM g^−1^ FW min^−1^.

### 4.9. Statistical Analyses

All statistical analyses were performed using multifactor analysis of variance (ANOVA/MANOVA) in Statistica 13 PL software (StatSoft, Tulsa, OK, USA). The graphs were plotted using means with standard error bars for each data point, and post-hoc comparisons were performed using Duncan’s multiple range test with a significance threshold of *p* ≤ 0.05.

Principal component analysis (PCA) was performed in Statistica software using the Principal Components and Classification Analysis (PCCA) procedure from the Multivariate Exploratory Techniques module. The analysis was based on the covariance matrix and conducted on a set of parameters measured after ten days of flooding in cold-acclimated plants. PCA was used to identify multivariate relationships between the two contrasting genotypes following cold acclimation and ten days of flooding. Separate analyses were performed for leaf and root datasets. The first two principal components were used for graphical visualization of sample distribution and variable loadings.

## 5. Conclusions

Cold acclimation alone did not differentiate the two rye genotypes in terms of frost tolerance. In contrast, flooding under low-temperature conditions of cold-acclimated plants enhanced freezing tolerance only in the pathogen-resistant genotype, indicating that flooding may have triggered mechanisms contributing to frost tolerance induction. This response was consistent with the moderate increases in *CBFIV* and *1-SST* transcript levels under flooding in line 343, suggesting a positive effect on the cold-response regulatory pathway or fructan metabolism. In contrast, although *CBFIV* transcript levels were similar in line 620, *1-SST* expression increased markedly after prolonged flooding, indicating an alteration in fructan metabolism under extended stress, which may have limited the induction of frost tolerance after flooding. Roots are the organs most strongly exposed to the combined effects of low temperature and flooding. In the susceptible line 620, this sensitivity may be associated with lower antioxidant capacity and the absence of the 50 kDa β-1,3-glucanase isoform, whereas the presence of this isoform in genotype 343 may be associated with improved frost tolerance. After ten days of flooding, the tolerant line 343 appeared to adopt a metabolic strategy characterized by increased cytosolic and membrane-bound SuS activity in the roots. In contrast, line 620 showed decreased membrane-bound SuS activity and no increase in cytosolic SuS activity, suggesting a limited capacity for sucrose turnover and potentially reflecting the progressive depletion of carbohydrate reserves under prolonged stress. Understanding these contrasting strategies provides valuable insight for breeding programs aimed at developing winter rye and other cereals with improved tolerance to combined cold and flooding stress in the context of climate change.

## Figures and Tables

**Table 1:** Snow mould resistance expressed as regrowth dry weight (% of the non-inoculated control). Significant differences between treatments are indicated by different letters (*p* ≤ 0.05).

	Regrowth Dry Weight (% of the Non-Inoculated Control)
343	51.30 a
620	11.93 b

**Table 2:** Reduced (GSH), oxidised (GSSG) and total glutathione content [µmol g^−1^ FW] in leaves and roots of two winter rye lines (343—resistant to snow mould, 620—susceptible to snow mould), cold acclimated and then subjected to one (CA 1F) or ten (CA 10F) days flooding stress under cold. Significant differences between treatments are indicated by different letters (*p* ≤ 0.05).

	Treatment	Genotype	GSH	GSSG	Total
leaves	CA	343	0.417 c	0.036 def	0.453 c
		620	0.344 d	0.049 cde	0.393 d
	CA 1F	343	0.463 b	0.051 cde	0.513 b
		620	0.465 b	0.040 def	0.504 b
	CA 10F	343	0.507 a	0.058 bcd	0.565 a
		620	0.528 a	0.069 abc	0.597 a
roots	CA	343	0.063 h	0.084 a	0.148 g
		620	0.024 i	0.077 ab	0.100 h
	CA 1F	343	0.259 f	0.021 f	0.280 f
		620	0.304 e	0.024 f	0.327 e
	CA 10F	343	0.164 g	0.018 f	0.182 g
		620	0.069 h	0.034 ef	0.103 h

**Table 3:** Genes, sequence origins, and designed primers and probes used in this study.

Gene Name	GenBnk ID	Forward Primer	Reverse Primer	Probe
*glu*-8	AM181312.1	GCCTCAACTACGCGACGTT	AGGTCAGCCCGTTGTTCTG	FAM-CAGCCGGGCACCACC-NFQ
1-*SST*	JQ728010.1	GTGCTGTACGTGCTCAAGGA	CCGGCCCAGCGAGTAC	FAM-ACGACCGGCACGACTG-NFQ
*TLP*3	AF099670.1	GACTTCTACGACATCTCGGTGATC	GGTGCTGCATGAGAAGTCCAT	FAM-ACGGCTTCAACCTTG-NFQ
*CBFIV*	EU194249.1	AGCGCGCTCTCCATCATC	CCATGCCGCCGATCCA	FAM-TCGCTGTCGAGCCCC-NFQ
*Actin*	EU153586.1	GTATCGCTGACCGTATGAGCAA	CGACGACCTTGATCTTCATGCT	FAM-CACCGCCCTTGCTCC-NFQ

## Data Availability

The original contributions presented in this study are included in the article. Further inquiries can be directed to the corresponding author.
